# Supplementary figures and images for: Patterns and associated factors of diabetes self-management: Results of a latent class analysis in a German population-based study
Source: PLoS One. 2021 Mar 19;16(3):e0248992. doi: 10.1371/journal.pone.0248992 (PMC7978380; doi:10.1371/journal.pone.0248992)

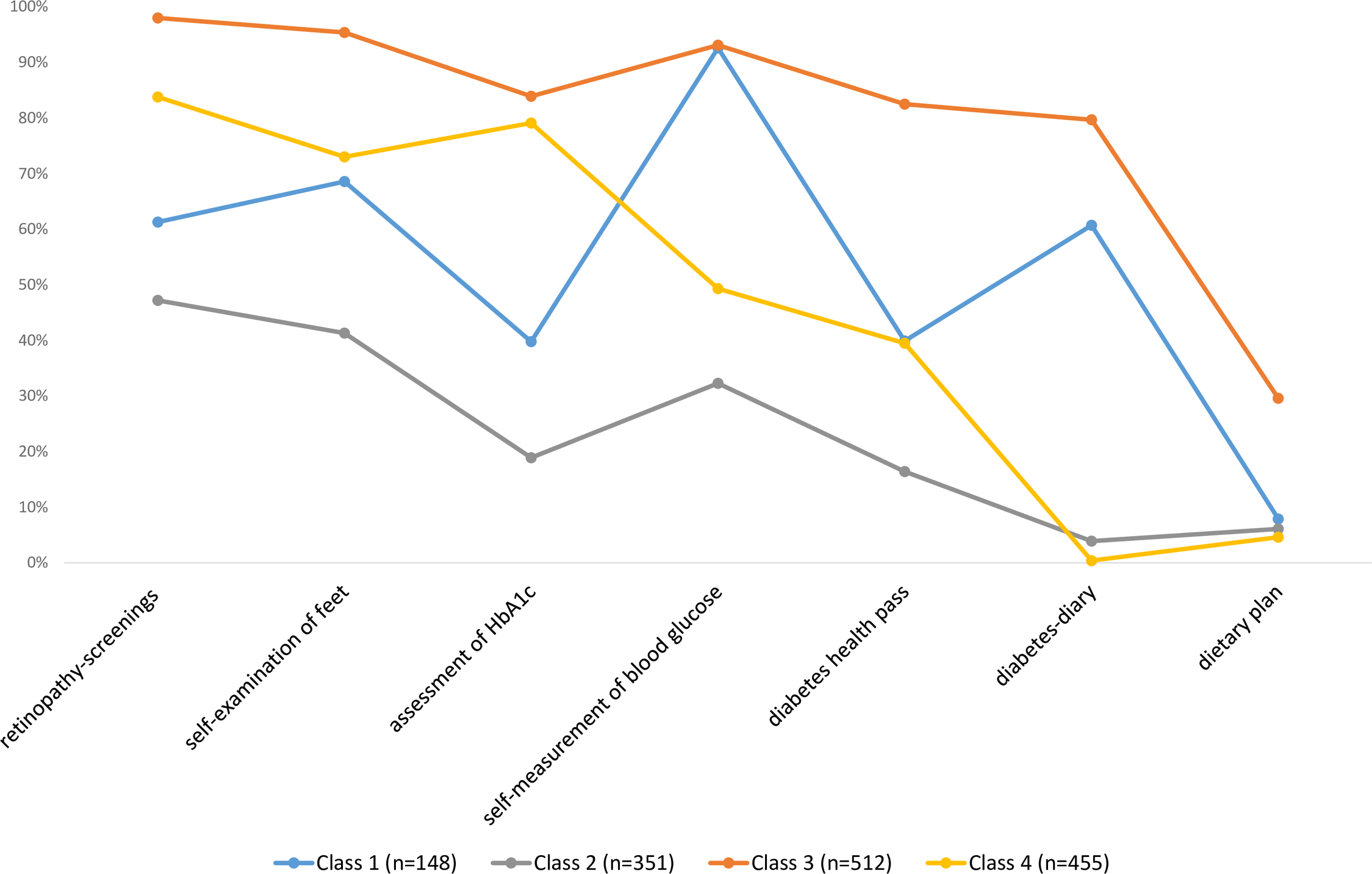

Supplement: S1 Fig — Conditional prevalences, n = 1,466. (TIF) [file pone.0248992.s001.tif]

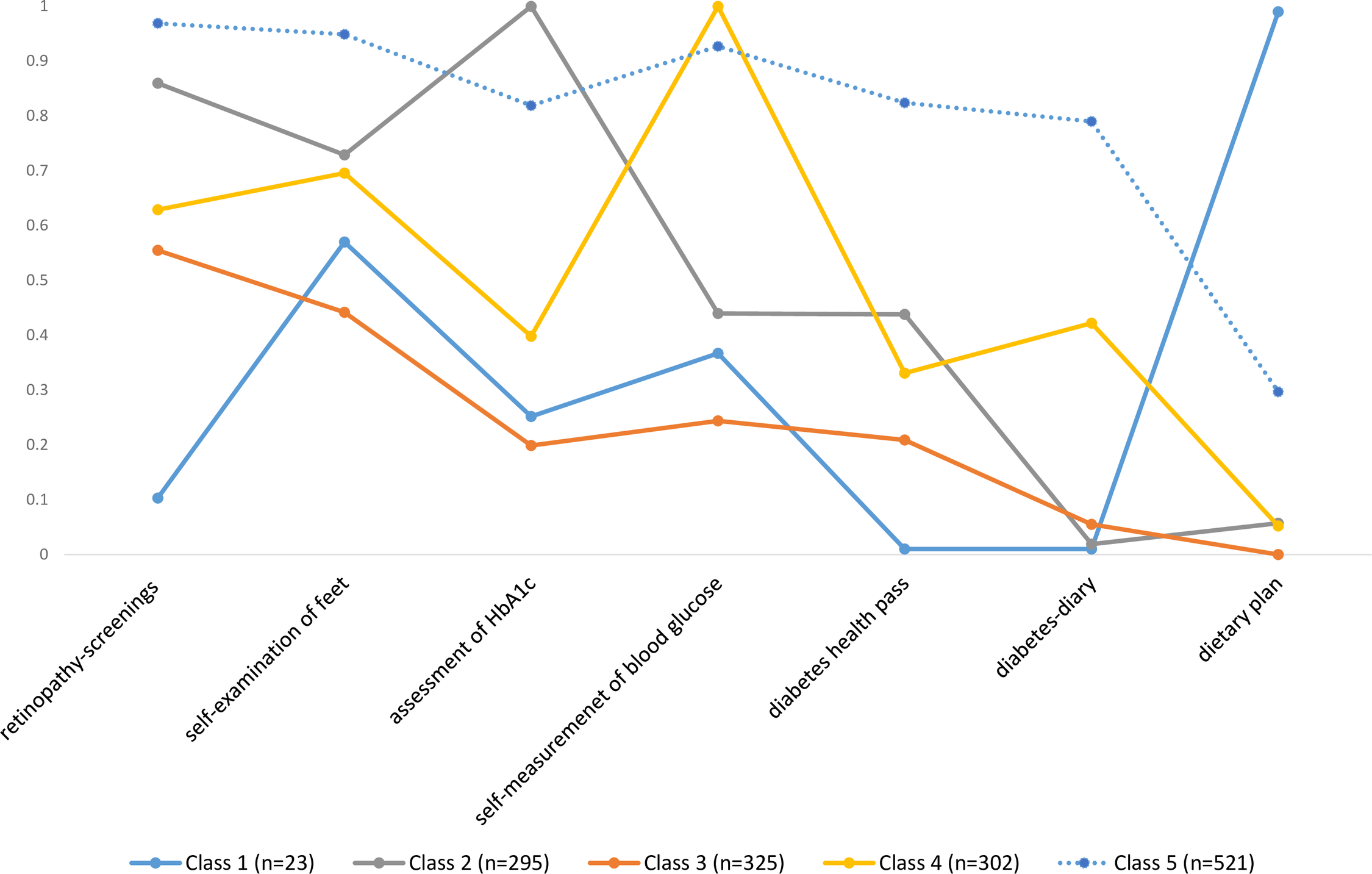

Supplement: S2 Fig — Conditional prevalences, n = 1,466. (TIF) [file pone.0248992.s002.tif]

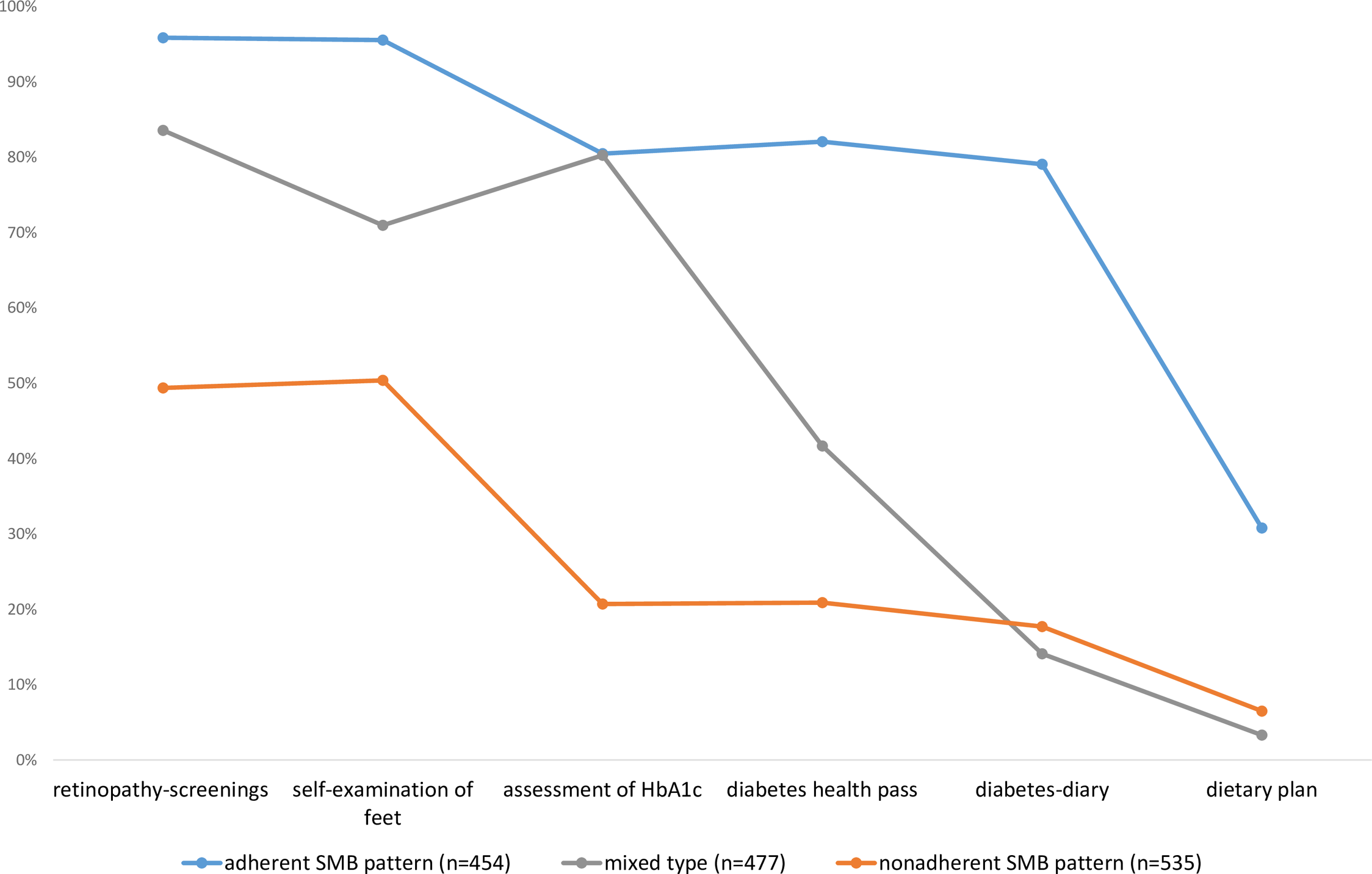

Supplement: S3 Fig — Conditional prevalences, n = 1,466. (TIF) [file pone.0248992.s003.tif]

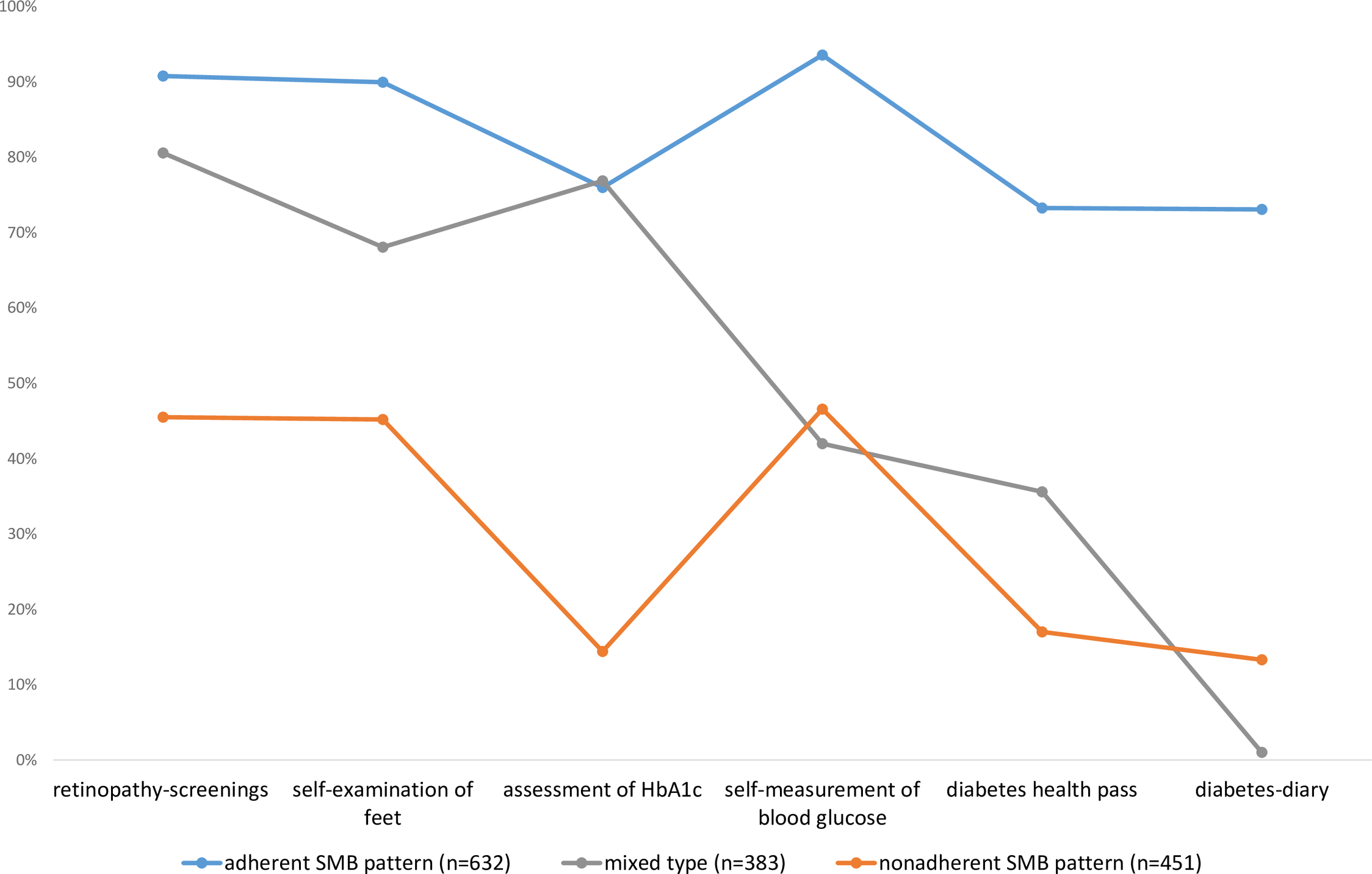

Supplement: S4 Fig — Conditional prevalences, n = 1,466. (TIF) [file pone.0248992.s004.tif]
