# Supplementary material for: Patterns and associated factors of diabetes self-management: Results of a latent class analysis in a German population-based study
Source: PLoS One. 2021 Mar 19;16(3):e0248992. doi: 10.1371/journal.pone.0248992 (PMC7978380; doi:10.1371/journal.pone.0248992)
Supplement: S1 Table — (DOCX) [file pone.0248992.s005.docx]

**S1 Table: Comparison of analysed net sample with complete cases (n=1,466) versus respondents excluded from the analysis due to missing values in exogenous covariates (n=241)**

|  | **cases excluded due to missing values (n=241)** | | **net sample of complete cases (n=1466)** | | ***p^c^*** |
| --- | --- | --- | --- | --- | --- |
|  | **n/n_valid_^a^** | **%** | **n/n_valid_^b^** | **%** |  |
| currently keeping dietary plan |  |  |  |  | 0.896 |
| *No* | 165 / 191 | 86.4% | 1,268 / 1,462 | 86.7% |  |
| *Yes* | 26 / 191 | 13.6% | 194 / 1,462 | 13.3% |  |
|  |  |  |  |  |  |
| currently keeping diabetes-diary |  |  |  |  | 0.596 |
| *No* | 128 / 191 | 67.0% | 952 / 1,463 | 65.1% |  |
| *Yes* | 63 / 191 | 33.0% | 511 / 1,463 | 34.9% |  |
|  |  |  |  |  |  |
| ever kept diabetes health pass |  |  |  |  | 0.725 |
| *No* | 99 / 190 | 52.1% | 781 / 1,461 | 53.5% |  |
| *Yes* | 91 / 190 | 47.9% | 680 / 1,461 | 46.5% |  |
|  |  |  |  |  |  |
| self-measurement of blood glucose |  |  |  |  | 0.519 |
| *Less than once a month* | 70 / 192 | 36.5% | 499 / 1,463 | 34.1% |  |
| *At least once a month* | 122 / 192 | 63.5% | 964 / 1,463 | 65.9% |  |
|  |  |  |  |  |  |
| self-examination of feet |  |  |  |  | 0.211 |
| *never* | 62 / 189 | 32.8% | 408 / 1,436 | 28.4% |  |
| *daily or occasionally* | 127 / 189 | 67.2% | 1,028 / 1,436 | 71.6% |  |
|  |  |  |  |  |  |
| retinopathy-screenings last 12 months |  |  |  |  | 0.677 |
| *never within last 12 months* | 45 / 199 | 22.6% | 349 / 1,457 | 24.0% |  |
| *at least once within last 12 months* | 154 / 199 | 77.4% | 1,108 / 1,457 | 76.0% |  |
|  |  |  |  |  |  |
| assessment of HbA1c |  |  |  |  | 0.689 |
| *< 4 times within last 12 months* | 74 / 184 | 40.2% | 598 / 1,432 | 41.8% |  |
| *at least 4 times within last 12 months* | 110 / 184 | 59.8% | 834 / 1432 | 58.2% |  |

This table shows absolute and relative frequencies not considering weighting factors.

a = We show unweighted absolute frequencies (n/nvalid);
b = We show unweighted relative frequencies (n/nvalid);
c - indicates test for significance between complete cases vs cases excluded due to missing values
Abbreviations: HbA1c – haemoglobin A1c; DSME – diabetes self-management education program
