# Supplementary material for: Patterns and associated factors of diabetes self-management: Results of a latent class analysis in a German population-based study
Source: PLoS One. 2021 Mar 19;16(3):e0248992. doi: 10.1371/journal.pone.0248992 (PMC7978380; doi:10.1371/journal.pone.0248992)
Supplement: S2 Table — (DOCX) [file pone.0248992.s006.docx]

**S2 Table: Wordings for various questions regarding SMB within the GEDA questionnaire**

| **SMB-Indicator** | **exact wording in GEDA-questionaire** | **Response categories** |
| --- | --- | --- |
| currently keeping dietary plan | Do you currently have a diet plan because of your diabetes? | Yes |
|  |  | No |
| currently keeping diabetes-diary | Are you currently keeping a diabetes diary? | Yes |
|  |  | No |
| ever kept diabetes health pass | Have you ever had a personal diabetes health pass? | Yes |
|  |  | No |
| self-measurement of blood glucose (SMBG) | Do you - or relatives for you - do perform blood glucose self-monitorings? If yes: How often do you - or relatives for you - check your blood glucose yourself? | No |
|  |  | daily / multiple times daily |
|  |  | x days per week (respondents can give a numerical response) |
|  |  | x times per Month (respondents can give a numerical response) |
| self-examination of feet | How often do you examine your feet for pressure points or open areas? | never |
|  |  | daily / multiple times daily |
|  |  | x times per week (respondents can give a numerical response) |
|  |  | x times per Month (respondents can give a numerical response) |
| retinopathy-screenings within last 12 months | In the last 12 months, how often have you had the back of your eye examined by an ophthalmologist (with dilated pupil drops)? | never |
|  |  | x times within the last 12 months (respondents can give a numerical response) |
| assessment of HbA1c | How often was your HbA1c, i.e. hemoglobin A1c, determined in the last 12 months? The blood value HBA1c is a specific value for the monitoring of diabetes. | never |
|  |  | x times within the last 12 months (respondents can give a numerical response) |
