# Supplementary material for: Patterns and associated factors of diabetes self-management: Results of a latent class analysis in a German population-based study
Source: PLoS One. 2021 Mar 19;16(3):e0248992. doi: 10.1371/journal.pone.0248992 (PMC7978380; doi:10.1371/journal.pone.0248992)
Supplement: S3 Table — (DOCX) [file pone.0248992.s007.docx]

**S3 Table. Results of the first sensitivity analysis (excluding self-measurement of blood glucose as indicator variable): Information criteria (penalized likelihood criteria) for a series of weighted Latent-Class models without covariates (unconditional models)**

| number of latent classes | Log-Likelihood | AIC | BIC | ABIC |
| --- | --- | --- | --- | --- |
| Independence | -5174.4 | 10360.8 | 10392.6 | 10373.5 |
| 2 | -4875.5 | 9777.0 | 9845.7 | 9804.4 |
| 3 | -4849.7 | 9739.4 | 9845.3 | 9781.7 |
| 4 | -4837.1 | 9728.2 | 9871.1 | 9785.3 |
| 5 | 4830.7 | 9729.3 | 9909.2 | 9801.2 |

*n=1466; all models took weighting factor into account;
Abbreviations: AIC – Akaike-Information-Criterion; BIC – Bayes-Information-Criterion; ABIC: sample-adjusted Bayes-Information-Criterion*
