# Supplementary material for: Patterns and associated factors of diabetes self-management: Results of a latent class analysis in a German population-based study
Source: PLoS One. 2021 Mar 19;16(3):e0248992. doi: 10.1371/journal.pone.0248992 (PMC7978380; doi:10.1371/journal.pone.0248992)
Supplement: S4 Table — (DOCX) [file pone.0248992.s008.docx]

**S4 Table. Results of the second sensitivity analysis (excluding dietary plan as indicator variable): Information criteria (penalized likelihood criteria) for a series of weighted Latent-Class models without covariates (unconditional models)**

| number of latent classes | Log-Likelihood | AIC | BIC | ABIC |
| --- | --- | --- | --- | --- |
| Independence | -5534.8 | 11081.7 | 11113.4 | 11094.3 |
| 2 | -5159.7 | 10345.4 | 10414.2 | 10372.9 |
| 3 | -5121.2 | 10282.5 | **10388.3** | 10324.7 |
| 4 | -5094.4 | 10242.8 | **10385.6** | **10299.8** |
| 5 | -5085.2 | **10238.4** | 10418.2 | 10310.2 |

*n=1466; all models took weighting factor into account;
Abbreviations: AIC – Akaike-Information-Criterion; BIC – Bayes-Information-Criterion; ABIC: sample-adjusted Bayes-Information-Criterion*
