# Supplementary material for: Patterns and associated factors of diabetes self-management: Results of a latent class analysis in a German population-based study
Source: PLoS One. 2021 Mar 19;16(3):e0248992. doi: 10.1371/journal.pone.0248992 (PMC7978380; doi:10.1371/journal.pone.0248992)
Supplement: S5 Table — (DOCX) [file pone.0248992.s009.docx]

**S5 Table. Results of the first sensitivity analysis (excluding self-measurement of blood glucose as indicator variable): Multinomial latent variable regressions of posterior probabilities predicted by sociodemographic and disease-related factors (logistic slopes and marginal effects, latent classes parameters fixed within Manual ML-three-step approach)**

|  |  | **logistic slope: mixed-type vs non-adherent SMB pattern** | |  | **logistic slope: adherent SMB pattern vs nonadherent SMB pattern** | |  | **marginal effects (average change or discrete change) ^a)^** | | |
| --- | --- | --- | --- | --- | --- | --- | --- | --- | --- | --- |
| **Modell** |  | **β** | **p** |  | **β** | **p** |  | **non adherent  SMB pattern** | **mixed-type** | **adherent  SMB pattern** |
| Modell 1 | ever-participation in DSME program (vs. never) | 1.628 | <0.001 |  | 3.054 | <0.001 |  | -47.9% | 9.5% | 38.4% |
|  | Intercept | -0.903 |  |  | -2.068 |  |  |  |  |  |
| Modell 2 | age (z-score) | 0.065 | 0.62 |  | 0.043 | 0.682 |  | -7.3% | 5.7% | 1.6% |
|  | Intercept | -0.081 |  |  | -0.085 |  |  |  |  |  |
| Modell 3 | female (vs. male) | 0.169 | 0.539 |  | 0.265 | 0.192 |  | -4.9% | 0.9% | 4.0% |
|  | Intercept | -0.157 |  |  | -0.204 |  |  |  |  |  |
| Modell 4 | SES-Score (z-Score) | -0.086 | 0.519 |  | -0.411 | <0.001 |  | 24.1% | 9.4% | -33.5% |
|  | Intercept | -0.085 |  |  | -0.151 |  |  |  |  |  |
| Modell 5 | living together (vs. not living together) | 0.170 | 0.591 |  | -0.218 | 0.323 |  | 0.7% | 5.9% | -6.6% |
|  | Intercept | -0.209 |  |  | 0.062 |  |  |  |  |  |
| Modell 6 | high/moderate limitation due illness (vs. none) | 0.080 | 0.77 |  | 0.593 | 0.004 |  | -7.6% | -4.4% | 12.0% |
|  | Intercept | -0.123 |  |  | -0.406 |  |  |  |  |  |
| Modell 7 | attendance toward health (z-Score) | 0.014 | 0.93 |  | 0.479 | <0.001 |  | -23.0% | -18.7% | 41.7% |
|  | Intercept | -0.084 |  |  | -0.118 |  |  |  |  |  |
| Modell 8 | employed (vs. unemployed / retired / unable) | -0.187 | 0.517 |  | -0.561 | 0.011 |  | 8.5% | 1.4% | -9.9% |
|  | Intercept | -0.026 |  |  | 0.068 |  |  |  |  |  |
| Modell 9 | time since diagnosis ≥ 10 years (vs. < 10 years) | 1.280 | 0.001 |  | 1.585 | <0.001 |  | -30.6% | 10.5% | 20.1% |
|  | Intercept | -0.593 |  |  | -0.776 |  |  |  |  |  |

**S5 Table (continued)**

|  |  | **logistic slope: mixed-type vs non-adherent SMB pattern** | |  | **logistic slope: adherent SMB pattern vs nonadherent SMB pattern** | |  | **marginal effects (average change or discrete change) ^a)^** | | |
| --- | --- | --- | --- | --- | --- | --- | --- | --- | --- | --- |
| **Modell** |  | **β** | **p** |  | **β** | **p** |  | **non-adherent  SMB pattern** | **mixed-type** | **adherent  SMB pattern** |
| multivariate Regression | ever-participation in DSME program (vs. never) | 1.674 | <0.001 |  | 3.082 | <0.001 |  | -47.6% | 11.7% | 35.9% |
|  | SES-Score (z-Score) | -0.104 | 0.539 |  | -0.458 | 0.003 |  | 23.5% | 9.9% | -33.4% |
|  | attendance toward health (z-Score) | -0.034 | 0.855 |  | 0.490 | 0.002 |  | -7.6% | -5.4% | 13.0% |
|  | employed (vs. unemployed / retired / unable) | -0.146 | 0.692 |  | -0.305 | 0.353 |  | 4.7% | -0.4% | -4.3% |
|  | time since diagnosis ≥ 10 years (vs. < 10 years) | 1.088 | 0.002 |  | 1.352 | <0.001 |  | -25.7% | 11.3% | 14.4% |
|  | Intercept | -1.319 |  |  | -2.682 |  |  |  |  |  |

*n=1466; notes: a) marginal effects for categorical, dichotomous predictors refer to differences in predicted posterior probabilities between the two possible values of the covariate (discrete change); marginal effects for metric predictors refer to the change in predicted posterior probabilities when covariate changes from minima to maxima
Abbreviations: DSME – structured education program for patients with diabetes mellitus, SES- socioeconomic status*
