# Supplementary material for: Patterns and associated factors of diabetes self-management: Results of a latent class analysis in a German population-based study
Source: PLoS One. 2021 Mar 19;16(3):e0248992. doi: 10.1371/journal.pone.0248992 (PMC7978380; doi:10.1371/journal.pone.0248992)
Supplement: S6 Table — (DOCX) [file pone.0248992.s010.docx]

**S6 Table. Results of the second sensitivity analysis (excluding dietary plan as indicator variable: Multinomial latent variable regressions of posterior probabilities predicted by sociodemographic and disease-related factors (logistic slopes and marginal effects, latent classes parameters fixed within Manual ML-three-step approach)**

|  |  | **logistic slope: mixed-type vs non-adherent SMB pattern** | |  | **logistic slope: adherent SMB pattern vs nonadherent SMB pattern** | |  | **marginal effects (average change or discrete change) ^a)^** | | |
| --- | --- | --- | --- | --- | --- | --- | --- | --- | --- | --- |
| **Modell** |  | **β** | **p** |  | **β** | **p** |  | **non adherent  SMB pattern** | **mixed-type** | **adherent  SMB pattern** |
| Modell 1 | ever-participation in DSME program (vs. never) | 1.027 | 0.001 |  | 3.281 | <0.001 |  | -41.2% | -13.1% | 54.3% |
|  | Intercept | -0.394 |  |  | -1.598 |  |  |  |  |  |
| Modell 2 | age (z-score) | 0.316 | 0.035 |  | -0.001 | 0.996 |  | -12.7% | 33.6% | -21.0% |
|  | Intercept | 0.005 |  |  | 0.491 |  |  |  |  |  |
| Modell 3 | female (vs. male) | 0.348 | 0.237 |  | 0.232 | 0.263 |  | -5.5% | 4.1% | 1.4% |
|  | Intercept | -0.143 |  |  | 0.392 |  |  |  |  |  |
| Modell 4 | SES-Score (z-Score) | -0.223 | 0.137 |  | -0.309 | 0.004 |  | 25.0% | -3.4% | -21.6% |
|  | Intercept | 0.003 |  |  | 0.468 |  |  |  |  |  |
| Modell 5 | living together (vs. not living together) | 0.102 | 0.759 |  | -0.016 | 0.945 |  | -0.6% | 2.2% | -1.7% |
|  | Intercept | -0.065 |  |  | 0.501 |  |  |  |  |  |
| Modell 6 | high/moderate limitation due illness (vs. none) | -0.003 | 0.99 |  | 0.348 | 0.085 |  | -4.2% | -4.4% | 8.6% |
|  | Intercept | 0.010 |  |  | 0.308 |  |  |  |  |  |
| Modell 7 | attendance toward health (z-Score) | 0.246 | 0.116 |  | 0.338 | 0.002 |  | -31.0% | 5.3% | 25.7% |
|  | Intercept | 0.035 |  |  | 0.510 |  |  |  |  |  |
| Modell 8 | employed (vs. unemployed / retired / unable) | -0.783 | 0.015 |  | -0.496 | 0.018 |  | 12.5% | -9.2% | -3.3% |
|  | Intercept | 0.240 |  |  | 0.651 |  |  |  |  |  |
| Modell 9 | time since diagnosis ≥ 10 years (vs. < 10 years) | 0.869 | 0.008 |  | 1.465 | <0.001 |  | -23.0% | -1.3% | 24.3% |
|  | Intercept | -0.302 |  |  | -0.138 |  |  |  |  |  |

**S6 Table (continued)**

|  |  | **logistic slope: mixed-type vs non-adherent SMB pattern** | |  | **logistic slope: adherent SMB pattern vs nonadherent SMB pattern** | |  | **marginal effects (average change or discrete change) ^a)^** | | |
| --- | --- | --- | --- | --- | --- | --- | --- | --- | --- | --- |
| **Modell** |  | **β** | **p** |  | **β** | **p** |  | **non adherent  SMB pattern** | **mixed-type** | **adherent  SMB pattern** |
| multi-variate Regress-ion | ever-participation in DSME program (vs. never) | 1.149 | 0.001 |  | 3.393 | <0.001 |  | -41.7% | -12.3% | 54.0% |
|  | SES-Score (z-Score) | -0.18 | 0.292 |  | -0.325 | 0.033 |  | 21.5% | 1.6% | -23.1% |
|  | attendance toward health (z-Score) | 0.129 | 0.402 |  | 0.327 | 0.028 |  | 8.7% | -18.2% | 9.5% |
|  | employed (vs. unemployed / retired / unable) | -0.655 | 0.087 |  | -0.461 | 0.162 |  | 10.8% | -8.5% | -2.3% |
|  | time since diagnosis ≥ 10 years (vs. < 10 years) | 0.597 | 0.078 |  | 1.109 | <0.001 |  | -15.8% | -2.1% | 18.0% |
|  | Intercept | -0.432 |  |  | -2.007 |  |  |  |  |  |

*n=1466; notes: a) marginal effects for categorical, dichotomous predictors refer to differences in predicted posterior probabilities between the two possible values of the covariate (discrete change); marginal effects for metric predictors refer to the change in predicted posterior probabilities when covariate changes from minima to maxima
Abbreviations: DSME – structured education program for patients with diabetes mellitus, SES- socioeconomic status*
